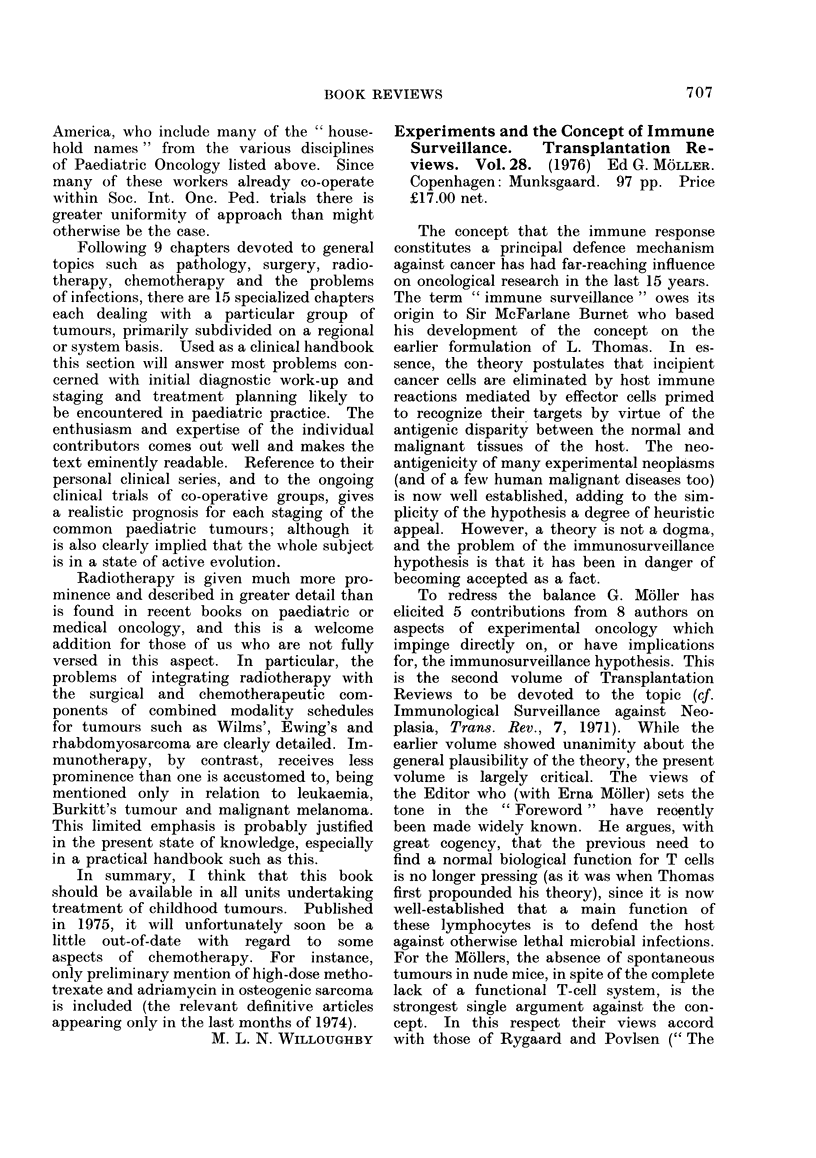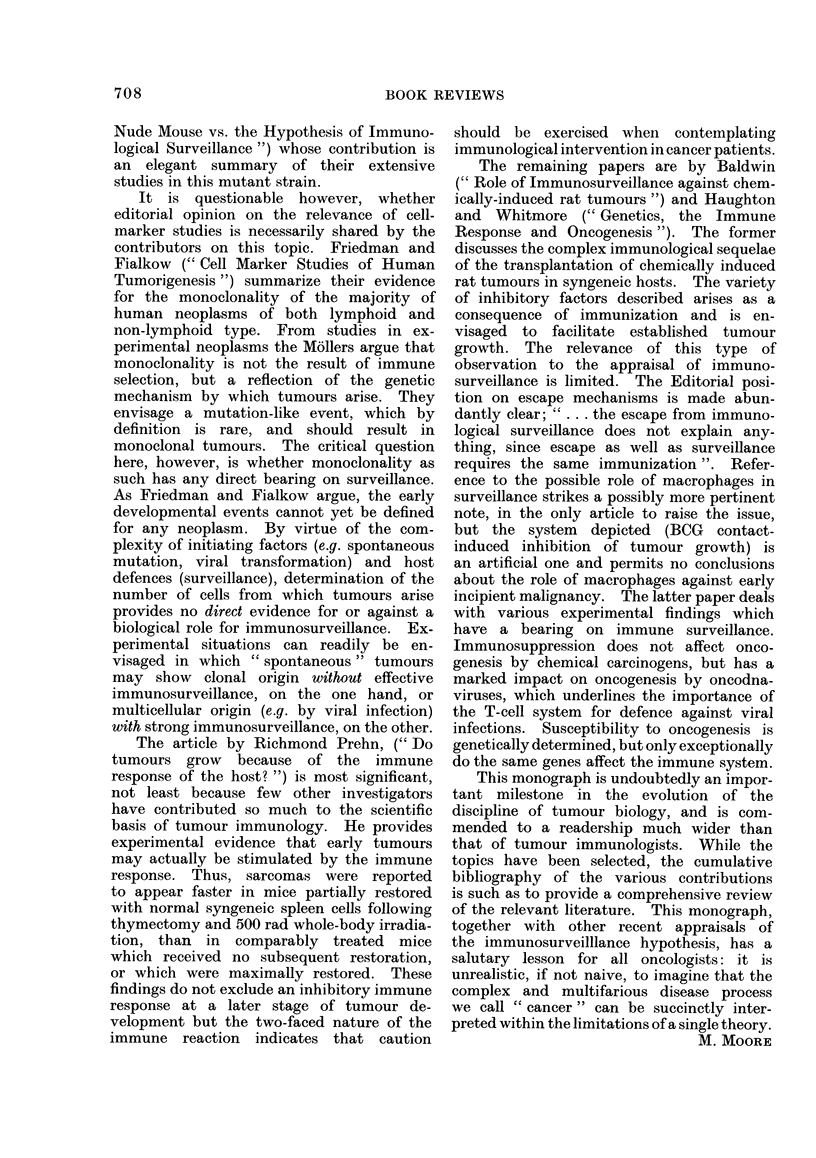# Experiments and the Concept of Immune Surveillance. Transplantation Re-views. Vol. 28

**Published:** 1977-05

**Authors:** M. Moore


**Experiments and the Concept of Immune Surveillance. Transplantation Reviews. Vol. 28.** (1976) Ed G. MÖLLER. Copenhagen: Munksgaard. 97 pp. Price £17.00 net.

The concept that the immune response constitutes a principal defence mechanism against cancer has had far-reaching influence on oncological research in the last 15 years. The term " immune surveillance " owes its origin to Sir McFarlane Burnet who based his development of the concept on the earlier formulation of L. Thomas. In essence, the theory postulates that incipient cancer cells are eliminated by host immune reactions mediated by effector cells primed to recognize their targets by virtue of the antigenic disparity between the normal and malignant tissues of the host. The neoantigenicity of many experimental neoplasms (and of a few human malignant diseases too) is now well established, adding to the simplicity of the hypothesis a degree of heuristic appeal. However, a theory is not a dogma, and the problem of the immunosurveillance hypothesis is that it has been in danger of becoming accepted as a fact.

To redress the balance G. Möller has elicited 5 contributions from 8 authors on aspects of experimental oncology which impinge directly on, or have implications for, the immunosurveillance hypothesis. This is the second volume of Transplantation Reviews to be devoted to the topic (*cf.* Immunological Surveillance against Neoplasia, *Trans. Rev.*, 7, 1971). While the earlier volume showed unanimity about the general plausibility of the theory, the present volume is largely critical. The views of the Editor who (with Erna Möller) sets the tone in the " Foreword " have recently been made widely known. He argues, with great cogency, that the previous need to find a normal biological function for T cells is no longer pressing (as it was when Thomas first propounded his theory), since it is now well-established that a main function of these lymphocytes is to defend the host against otherwise lethal microbial infections. For the Möllers, the absence of spontaneous tumours in nude mice, in spite of the complete lack of a functional T-cell system, is the strongest single argument against the concept. In this respect their views accord with those of Rygaard and Povlsen (" The

Nude Mouse vs. the Hypothesis of Immunological Surveillance ") whose contribution is an elegant summary of their extensive studies in this mutant strain.

It is questionable however, whether editorial opinion on the relevance of cell-marker studies is necessarily shared by the contributors on this topic. Friedman and Fialkow (" Cell Marker Studies of Human Tumorigenesis ") summarize their evidence for the monoclonality of the majority of human neoplasms of both lymphoid and non-lymphoid type. From studies in experimental neoplasms the Möllers argue that monoclonality is not the result of immune selection, but a reflection of the genetic mechanism by which tumours arise. They envisage a mutation-like event, which by definition is rare, and should result in monoclonal tumours. The critical question here, however, is whether monoclonality as such has any direct bearing on surveillance. As Friedman and Fialkow argue, the early developmental events cannot yet be defined for any neoplasm. By virtue of the complexity of initiating factors (*e.g.* spontaneous mutation, viral transformation) and host defences (surveillance), determination of the number of cells from which tumours arise provides no *direct* evidence for or against a biological role for immunosurveillance. Experimental situations can readily be envisaged in which " spontaneous " tumours may show clonal origin *without* effective immunosurveillance, on the one hand, or multicellular origin (*e.g.* by viral infection) *with* strong immunosurveillance, on the other.

The article by Richmond Prehn, (" Do tumours grow because of the immune response of the host? ") is most significant, not least because few other investigators have contributed so much to the scientific basis of tumour immunology. He provides experimental evidence that early tumours may actually be stimulated by the immune response. Thus, sarcomas were reported to appear faster in mice partially restored with normal syngeneic spleen cells following thymectomy and 500 rad whole-body irradiation, than in comparably treated mice which received no subsequent restoration, or which were maximally restored. These findings do not exclude an inhibitory immune response at a later stage of tumour development but the two-faced nature of the immune reaction indicates that caution should be exercised when contemplating immunological intervention in cancer patients.

The remaining papers are by Baldwin (" Role of Immunosurveillance against chemically-induced rat tumours ") and Haughton and Whitmore (" Genetics, the Immune Response and Oncogenesis "). The former discusses the complex immunological sequelae of the transplantation of chemically induced rat tumours in syngeneic hosts. The variety of inhibitory factors described arises as a consequence of immunization and is envisaged to facilitate established tumour growth. The relevance of this type of observation to the appraisal of immunosurveillance is limited. The Editorial position on escape mechanisms is made abundantly clear; " . . . the escape from immunological surveillance does not explain anything, since escape as well as surveillance requires the same immunization ". Reference to the possible role of macrophages in surveillance strikes a possibly more pertinent note, in the only article to raise the issue, but the system depicted (BCG contact-induced inhibition of tumour growth) is an artificial one and permits no conclusions about the role of macrophages against early incipient malignancy. The latter paper deals with various experimental findings which have a bearing on immune surveillance. Immunosuppression does not affect oncogenesis by chemical carcinogens, but has a marked impact on oncogenesis by oncodnaviruses, which underlines the importance of the T-cell system for defence against viral infections. Susceptibility to oncogenesis is genetically determined, but only exceptionally do the same genes affect the immune system.

This monograph is undoubtedly an important milestone in the evolution of the discipline of tumour biology, and is commended to a readership much wider than that of tumour immunologists. While the topics have been selected, the cumulative bibliography of the various contributions is such as to provide a comprehensive review of the relevant literature. This monograph, together with other recent appraisals of the immunosurveilllance hypothesis, has a salutary lesson for all oncologists: it is unrealistic, if not naive, to imagine that the complex and multifarious disease process we call " cancer " can be succinctly interpreted within the limitations of a single theory.

M. MOORE